# Effect of commercial rye whole-meal bread on postprandial blood glucose and gastric emptying in healthy subjects

**DOI:** 10.1186/1475-2891-8-26

**Published:** 2009-06-16

**Authors:** Joanna Hlebowicz, Jenny Maria Jönsson, Sandra Lindstedt, Ola Björgell, Gassan Darwich, Lars-Olof Almér

**Affiliations:** 1 Department of Medicine, University of Lund, Malmö University Hospital, Malmö, Sweden; 2 Department of Oncology, Lund University Hospital, Lund, Sweden; 3 Department of Cardiothoracic Surgery, Lund University Hospital, Lund, Sweden; 4 Department of Radiology, University of Lund, Malmö University Hospital, Malmö, Sweden

## Abstract

**Background:**

The intake of dietary fibre has been shown to reduce the risk of developing diabetes mellitus. The aim of this study was to compare the effects of commercial rye whole-meal bread containing whole kernels and white wheat bread on the rate of gastric emptying and postprandial glucose response in healthy subjects.

**Methods:**

Ten healthy subjects took part in a blinded crossover trial. Blood glucose level and gastric emptying rate (GER) were determined after the ingestion of 150 g white wheat bread or 150 g whole-meal rye bread on two different occasions after fasting overnight. The GER was measured using real-time ultrasonography, and was calculated as the percentage change in antral cross-sectional area 15 and 90 minutes after completing the meal.

**Results:**

No statistically significant difference was found between the GER values or the blood glucose levels following the two meals when evaluated with the Wilcoxon signed rank sum test.

**Conclusion:**

The present study revealed no difference in postprandial blood glucose response or gastric emptying after the ingestion of rye whole-meal bread compared with white wheat bread.

**Trial registration:**

NCT00779298

## Background

Evidence has been presented showing that changing the diet can control the blood glucose level and help prevent the development of type 2 diabetes. The American Diabetes Association recommends an increased intake of dietary fibre and whole grain products to prevent the development of type 2 diabetes and cardiovascular disease [1]. Cereals are the most important source of dietary fibre throughout the world, and bread is an essential part of the Swedish diet. The fibre in cereals is located mainly in the outer layer of the kernels. The term "whole grain" is often used to describe both whole-meal products in which the structure of the kernel has been destroyed and cereal products in which a large proportion of the grain is intact. However, there seems to be a major difference in metabolic response between whole grain and whole-meal products. Whole kernels appear to be more effective in reducing glucose response than dietary fibre [2,3]. The preparation, cooking and particle size may also affect the metabolic response. The germ of the whole grain acts as a natural amylase inhibitor, which can be destroyed during the milling of wheat into whole-meal flour [4]. There are many definitions of dietary fibre, including lignin and a range of polysaccharides derived from cell walls that are poorly digested in the upper intestine. In an attempt to explain the physiological effects of different types of fibre, dietary fibre has been divided into two groups, soluble and insoluble. Soluble dietary fibre is partially but not entirely water-soluble, and includes pectin, guar gum (galactomannan) and glucomannan (also known as konjac mannan), psyllium, β-glucan and arabinoxylans. Rye contains more soluble fibre in the form of β-glucan and arabinoxylans than wheat [5]. Gastric emptying, among other factors, regulates the postprandial blood glucose response, and a delay in gastric emptying is associated with a lower postprandial blood glucose level. It has been suggested that lower postprandial glucose and insulin levels observed after the consumption of different combinations of dietary fibre could be caused by reduced GER, mouth-to-caecum transit or delayed absorption of glucose in the small intestine. Previous studies on the effects of dietary fibre show divergent effects on the GER [6-12]. Another hypothesis that has been proposed is that dietary fibre is fermented in the colon by the bacterial flora, leading to the release of short-chain fatty acids, lowering postprandial glucose levels [13]. It has also been shown that the postprandial insulin response of healthy subjects to whole-meal rye bread and rye bread with whole kernels was lower than that to white bread, while the glucose response was not affected [13,14]. Neither was the GER affected when measured indirectly with paracetamol [14]. The paracetamol method is dependent on the release and absorption of paracetamol across the small intestine, which makes this method unreliable, as the pharmacokinetics of paracetamol varies within and between individuals [15,16]. The aim of this study was thus to compare the effects of whole-meal rye bread containing whole kernels with those of white wheat bread on the postprandial blood glucose response and the GER.

## Method

Ten healthy subjects, seven women and three men; mean age 26 ± 1 years [range 23–35 years]; mean BMI 24.1 ± 0.8 kg/m^2 ^[range 21.0–27.7 kg/m^2^], without symptoms or a prior history of gastrointestinal disease, abdominal surgery or diabetes mellitus, were included in the study. The subjects were recruited from the population in Skåne, the southernmost county of Sweden. None of the subjects was a smoker or a snuff user. One of the subjects was taking hypertension medication and one levothyroxine. None of the subjects used any drugs on the day of the examination. The subjects were examined between 8:00 and 10:00 after fasting for 8 hours. Each subject was required to have a normal fasting blood glucose level on the day of the study. If a subject showed gastrointestinal tract symptoms (diarrhoea or constipation) the study was postponed.

Each test meal was intended to contain 50 g carbohydrates from the test bread, ham and 300 ml FUN Light fruit drink (caloric value 3 kcal, 0.6 g carbohydrates) (Procordia Food AB, 241 81 Eslöv, Sweden), ingested over a period of 10 min. The nutrient composition of the test bread was not analysed at the start of the study, and the portions of bread were calculated based on data from the bakeries. According to the bakery (Cerealia Bakeries AB, Lund, Sweden) the reference white wheat bread (*Storform*) contained 34.65% available carbohydrates, while the rye whole-meal bread (*Danskt rågbröd*) containing whole kernels (Hemköpskedjan AB, Solna, Sweden) contained 41.8% available carbohydrates. The content of available carbohydrates was analysed according to Holm et al. [17]. The contents of dietary fibre, fat and proteins given in product information were assumed to be correct (Table 1). The meals were served in a random order at intervals of 1 week. Randomization was performed using a table of random numbers. The study was carried out during a period of 8 weeks.

**Table 1:** Nutrient composition of the two kinds of bread.

	Portion size (g)	Available CHO (g)	Dietary fibre (g)	Protein (g)	Fat (g)	Energy content (kJ)
White bread	150	52.0	0	13.5	0	435
Rye whole-meal bread	150	62.7	3.75	12.75	4.5	390

The GER was estimated using a previously described ultrasound method [18]. The examinations were performed using two different kinds of ultrasound equipment (Siemens Acuson Sequoa 512, and Siemens Elegra, Siemens Medical Solutions, Mountains View, CA, USA) with an abdominal 2.5–4 MHz transducer. However, all values of the GER were calculated using data collected with the same piece of equipment. The measurements of the diameter of the gastric antrum were performed by the same radiologist, who was blinded with regard to the meals. The measurements were made 15 and 90 minutes after the end of the meal. The GER was expressed as the percentage change in the antral cross-sectional area between 15 and 90 min after the meal.

Finger-prick capillary samples were collected after fasting and 40, 60 and 90 min after the end of the meal to measure blood glucose levels. Blood glucose concentrations were measured with a HemoCue Glucose system (HemoCue AB, Ängelholm, Sweden). The change in blood glucose level was calculated as the difference between the blood glucose level before the meal (fasting value) and 40, 60 and 90 min after the end of the meal. The blood glucose was determined from the areas under the curves (AUCs), using the area above zero, for each subject (Graph Pad PRISM, version 4, San Diego). These values are presented as the mean ± SEM for the whole group.

All statistical calculations were performed using SPSS for Windows software (version 14.0, 2005). Differences in the GER, gastric antral cross-sectional area and blood glucose levels were evaluated with the Wilcoxon signed rank sum test. Values of p < 0.05 were considered significant.

The study was performed according to the Helsinki declaration. All subjects gave written, informed consent before participating in the experiments.

## Results

### Postprandial blood glucose response

**Figure 1:**
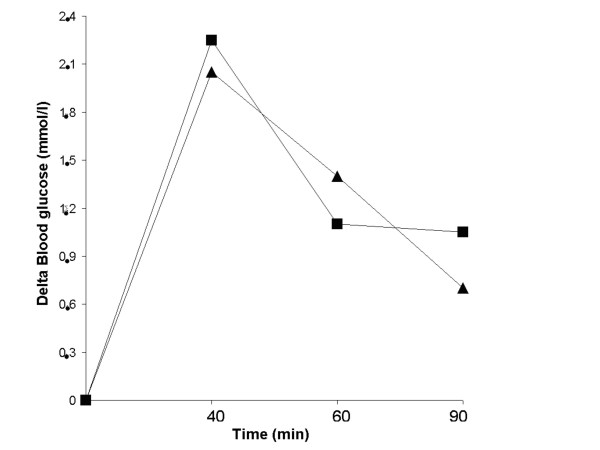
**The mean change in blood glucose concentration in ten healthy subjects after the ingestion of meals containing rye whole-meal bread (triangle), and white wheat bread (square)**. No significant differences were found between the various meals according to the Wilcoxon signed rank sum test.

The mean fasting blood glucose level before the ingestion of white bread was 4.4 ± 0.1 mmol/l and was not significantly different from that before the ingestion of rye whole-meal bread, which was 4.3 ± 0.1 mmol min/l. No significant differences were seen in blood glucose response at different times, or in the incremental areas under the postprandial glucose curves between the different bread meals (Figure 1). The mean AUCs after ingestion of white bread and rye whole-meal bread were 121.1 ± 17.9, and 125.2 ± 21.2 mmol min/l, respectively.

### Gastric emptying rate

**Figure 2:**
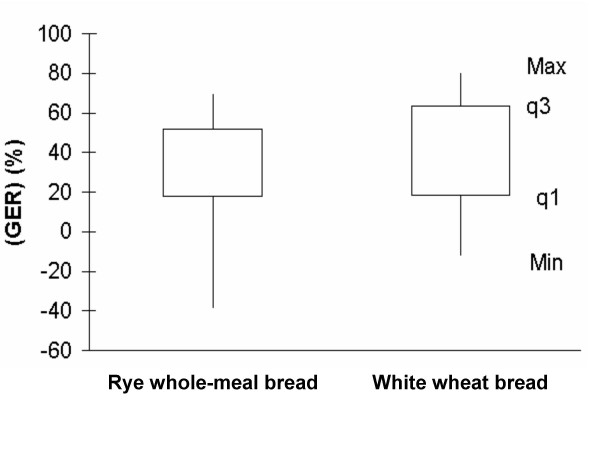
**Gastric emptying rate following the ingestion of rye whole-meal bread and white wheat bread, in ten healthy subjects**. The median, minimum (Min) and maximum (Max) values, and the values of the first (q1) and the third (q3) quartiles are shown. No significant differences were found between the GERs according to the Wilcoxon signed rank sum test.

No significant differences were observed between the meals with regard to GER (Figure 2). The median value of the GER after the reference meal of white bread was estimated to be 37% (q1 = 10%, q3 = 66%) while the corresponding value after the rye whole-meal bread meal was estimated to be 45% (q1 = 9%, q3 = 54%). The median values of the antral cross-sectional area after the ingestion of the white bread meal 15 and 90 min after the end of the meal were 963 mm^2 ^(q1 = 523 mm^2^, q3= 1110 mm^2^) and 523 mm^2 ^(q1 = 297 mm^2^, q3 = 759 mm^2^), respectively. After the ingestion of the rye whole-meal bread the corresponding values were 660 mm^2 ^(q1 = 427 mm^2^, q3= 957 mm^2^) and 416 mm^2 ^(q1= 312 mm^2^, q3= 585 mm^2^). The median gastric antral cross-sectional area was thus significantly larger after ingestion of the white bread than after ingestion of the rye whole-meal bread at 15 min (p = 0.047). However, there was no significant difference between gastric antral cross-sectional areas at 90 min.

## Discussion

The aim of this study was to elucidate the effects of dietary fibre and whole kernels present in rye-based bread on gastric emptying rate and glycaemic response in healthy subjects. Our hypothesis was that dietary fibre would lower the postprandial blood glucose response, in comparison with the reference meal, due to delayed gastric emptying. We were not able to verify this hypothesis. The rye whole-meal bread meal contained a higher total amount of available carbohydrates, 62.7 g, than the white bread meal, which contained 52.0 g. Unfortunately, the amount of available carbohydrates in the bread was not available at the start of the study, and the portions of the bread were calculated using data supplied by the bakeries. It would have been better to determine the test portions as grams of bread rather than available carbohydrates, since people usually eat a certain number of slices of bread. The rye whole-meal bread had a lower total caloric value (390 kcal) than the white bread (435 kcal). It has been found previously that the GER is lower following a meal with a higher caloric value [19]. Still, the difference in the amount of fibre was essential. Despite their different fibre contents and botanical structure, the rye whole-meal bread and the white bread showed similar postprandial glycaemic responses in this study. The rye kernels in the whole-meal bread may have been disrupted during the baking process, thus increasing the enzymatic accessibility of the starch. Unfortunately, neither the structure of the bread nor the amount of soluble fibre was investigated in the present study. The blood glucose levels were not measured very frequently during this study, and the glucose peak concentration may have been missed in some of the subjects. However, the lack of difference in GER and postprandial blood glucose response between the different kinds of bread is in agreement with studies performed by Juntunen et al. and Leinonen et al., who compared the effects of whole-kernel rye bread and whole-meal rye bread to white wheat bread in healthy subjects [10,14,20]. The ingestion of rye bread has been found to induce lower postprandial insulin levels, and glucose-dependent insulinotropic polypeptide (GIP) than wheat bread [10,20]. Furthermore, a lower C-peptide response after ingestion of rye bread indicates a reduced pancreatic secretion of insulin [20]. Unfortunately we did not measure insulin, GIP or C-peptide levels in this study. Dietary fibre and resistant starch may be fermented in the colon by the bacterial flora, leading to the release of short-chain fatty acids which may improve the glucose tolerance to the following meal [13]. There may thus have been a second-meal effect not evaluated in the present study.

A relationship between antral area and satiety in healthy subjects has been observed by others [21-25]. A limitation of this study is that we did not evaluate the satiety. The median antrum area 15 min after the intake of the white bread meal was significantly larger than that after the intake of the rye whole-meal bread meal. This is probably due to the larger volume of the white bread than the rye whole-meal bread. The negative values of GER are due to larger gastric antral cross-sectional areas at 90 min, which could be due to an increase in the amount of gastric juices and saliva in the stomach.

## Conclusion

In the present study, no difference was found in postprandial blood glucose response or gastric emptying after the ingestion of rye whole-meal bread compared with white bread.

## Competing interests

The authors declare that they have no competing interests.

## Authors' contributions

The authors' contributions were as follows: JMJ, GD, JH, and LOA contributed to the design of the study; JMJ was responsible for recruiting the subjects and carried out the practical aspects of the study. OB performed the ultrasound examinations; JH, SL, and JMJ conducted the statistical calculations; JH created the graphs. JH wrote the first draft of the manuscript and, GD, JMJ, SL, OB, and LOA made critical revisions of the manuscript. All authors read and approved the final manuscript.
